# Delayed Migration of an Endovascular Coil to the Right Ventricle Managed by Minimally Invasive Surgical Removal Following the Treatment of Pelvic Congestion Syndrome

**DOI:** 10.7759/cureus.108006

**Published:** 2026-04-30

**Authors:** Maikel den Breejen, Tom Welter, Eveline Patteet, Bernard Paelinck, Inez Rodrigus

**Affiliations:** 1 Cardiac Surgery, Antwerp University Hospital, Edegem, BEL; 2 Cardiology, Antwerp University Hospital, Edegem, BEL

**Keywords:** coil migration, endovascular coil embolization, minimal invasive cardiac surgery, pelvic congestion syndrome, right ventricle, tricuspid valve

## Abstract

Ovarian vein coil embolization is a well-established treatment for pelvic congestion syndrome. Although regarded as a safe and effective procedure, complications related to coil migration to the heart remain exceedingly rare but require a multidisciplinary approach to weigh the risks and benefits of conservative management against surgical intervention. Our case describes an asymptomatic delayed coil migration to the right ventricle attached to the subvalvular apparatus observed on echocardiography. Considering the risk of an endovascular retrieval and to mitigate further risks of complications, the coil was successfully removed via a minimally invasive right mini-thoracotomy.

## Introduction

Pelvic congestion syndrome is a cause of chronic pelvic pain in women of reproductive age. Patients typically present with non-cyclic pelvic pain exacerbated by prolonged standing, menstruation, or intercourse. It is often linked to dyspareunia, dysmenorrhea, urinary issues, or vulvar varicosities [1]. While the etiology is multifactorial and not fully understood, contributing mechanisms include hormonal influences, venous valve insufficiency, external venous obstruction, and anatomical variants.

Initial management includes pelvic floor physical therapy, cognitive-behavioral therapy, and ovarian suppression or vasoconstrictive medication. Although helpful in the short term, long-term pharmacological treatment is not recommended due to potential side effects [2].

In patients refractory to conservative management, transvenous embolization of the ovarian and iliac veins has emerged as an effective treatment modality due to its relative safety, effectiveness, and durability compared to surgical options [3,4]. It offers high technical success rates and symptom improvement in 82.1%-100% of patients [5]. Surgical interventions, such as open retroperitoneal or laparoscopic ovarian vein ligation, should only be considered if embolization is unsuccessful.

Despite its safety and efficacy, ovarian vein embolization is not without risks. Reported adverse events occur in approximately 0.85%-10% of cases and include access-site hematoma, contrast reactions, deep venous thrombosis, and post-embolization syndrome [5]. However, coil migration to the heart is extremely rare. 

Device-related complications, such as coil protrusion and migration, are clinically significant and can be potentially life-threatening, particularly when there is embolization to the right heart or pulmonary vasculature [4,6,7]. Identified risk factors for coil migration include large veins (>12 mm), undersized coils, and deployment in proximity to central venous outflow [8]. Intraoperative migration can be immediately treated using a snare technique, but delayed migration poses risks such as thrombosis, arrhythmias, pulmonary infarction, and systemic embolism [9].

Management strategies described in literature vary depending on clinical presentation and coil location, ranging from conservative management and observation to endovascular retrieval or open surgical intervention [10-14]. However, due to the rarity of this complication, evidence remains limited, and a clear consensus regarding optimal management is lacking.

We report a case of delayed coil migration to the right ventricle for which surgical removal was performed via a minimally invasive approach after endovascular retrieval was deemed unfeasible. This case highlights an alternative surgical strategy in the management of intracardiac coil migration and contributes to the limited body of literature guiding decision-making in this rare but serious complication.

## Case presentation

A 34-year-old woman was referred to the cardiac surgery department following transvenous embolization of the left ovarian vein for pelvic congestion syndrome. The procedure, using a right internal jugular approach, involved coil embolization of the left ovarian and iliac veins with spirales and fibered pushable coils (Angiocare BV, Amersfoort, Netherlands) supplemented with n-hexyl cyanoacrylate. Her initial symptoms included chronic lower abdominal and pelvic pain.

**Figure 1:**
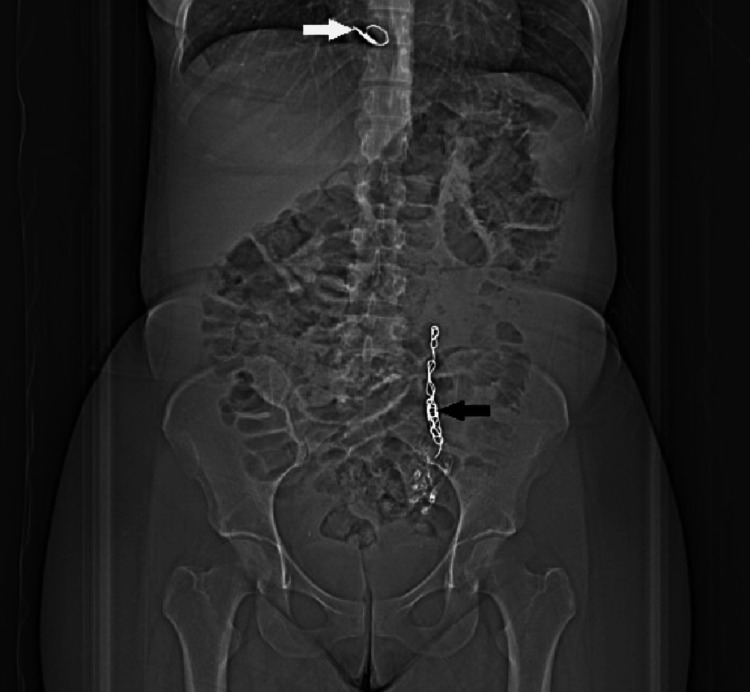
Plain radiography showing the endovascular coil positioned in the right ventricle after ovarian vein embolization The white arrow indicates the coil in the right ventricle and the black arrow indicates coils in the left ovarian vein.

One week post procedure, she presented to the emergency department with left abdominal pain and fever. Abdominal computed tomography confirmed successful sealing of the venous plexus, but plain chest radiography revealed migration of the distal fibered coil to the right ventricle (Figure 1).

Transthoracic and transesophageal echocardiography localized the coil in the right ventricular cavity, attached to the subvalvular apparatus of the tricuspid valve at the level of the posterior leaflet, adherent to the ventricular wall. There was no visible thrombus formation (Video 1).

**Video 1:** Apical transthoracic echocardiogram focused on the right ventricular cavity displaying mobile endovascular coil in the subvalvular apparatus of the tricuspid valve

Transvenous angiography demonstrated motion of the coil during the cardiac cycle, suggesting partial fixation within the myocardium (Video 2).

**Video 2:** Angiography showing the motion of the coil with the cardiac cycle, suggesting ingrowth into the ventricular wall

Given its attachment to the subvalvular apparatus of the tricuspid valve and its apparent ingrowth, endovascular retrieval was deemed too risky due to the potential for tricuspid valve injury and right ventricular perforation. Following multidisciplinary heart team discussion, surgical removal was favored over conservative management to mitigate risks of infective endocarditis, valvular dysfunction, right ventricular wall perforation, and further embolization.

**Figure 2:**
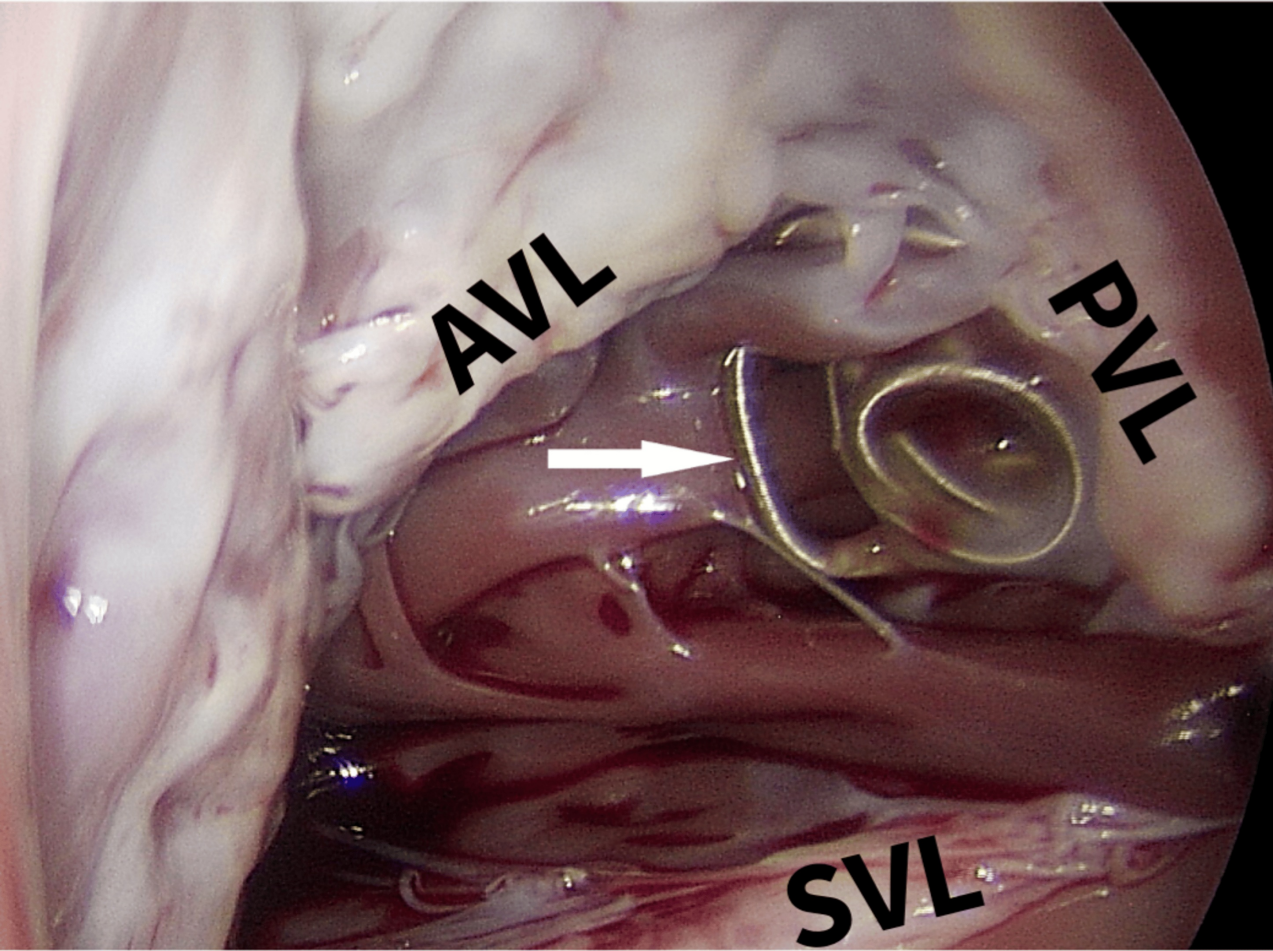
Intraoperative view of the tricuspid valve with the coil visible behind the PVL AVL: anterior valve leaflet; PVL: posterior valve leaflet; SVL: septal valve leaflet The white arrow indicates the migrated coil.

Surgical extraction was performed via a right mini-thoracotomy in the fifth intercostal space. Cardiopulmonary bypass was established through femoral cannulation, followed by aortic cross-clamping and administration of antegrade blood cardioplegia. Through a right atriotomy, the coil was identified underneath the tricuspid valve at the junction of the septal and posterior leaflet (Figure 2).

**Figure 3:**
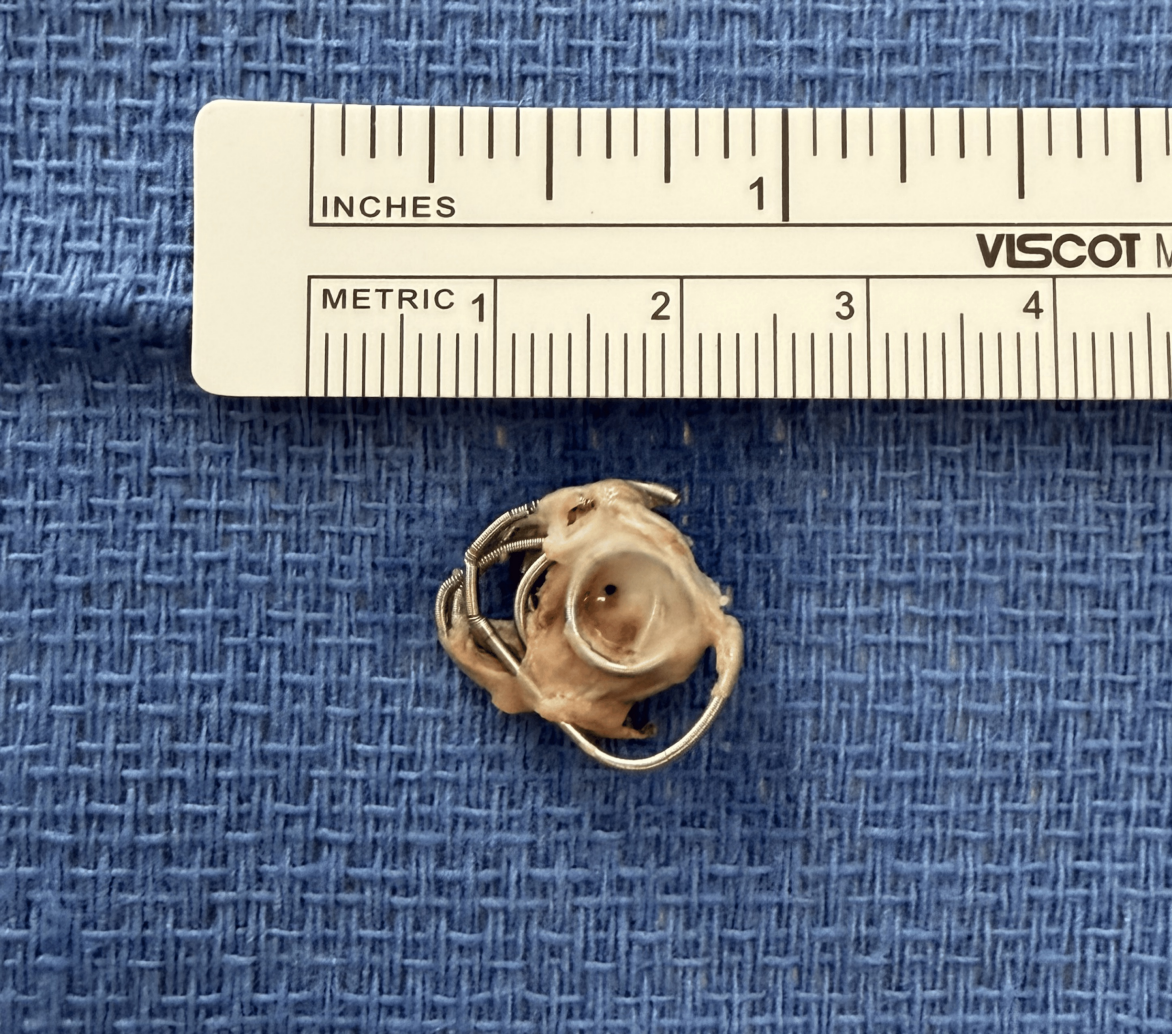
Retrieved coil from the right ventricle, measuring approximately 1.5 cm x 1.5 cm

Grasping of the coil demonstrated partial ingrowth into the myocardium. The coil was successfully removed without injury to the tricuspid valve apparatus or the right ventricular wall (Figure *3*).

Aortic cross-clamp and cardiopulmonary bypass times were 24 and 35 minutes, respectively.

The postoperative course was uneventful. The patient was discharged on postoperative day four. Follow-up transthoracic echocardiography demonstrated preserved right ventricular function without significant tricuspid regurgitation.

## Discussion

In the present case, the coil was located in the right ventricle, positioned below the tricuspid valve and adherent to the ventricular wall, with evidence of dynamic motion during the cardiac cycle. These features suggested partial myocardial ingrowth and posed a significant risk of tricuspid valve injury or right ventricular perforation if endovascular retrieval was attempted. Given these anatomical findings, surgical removal was deemed the safest strategy following multidisciplinary evaluation.

While surgical extraction has traditionally been performed via median sternotomy [10,15], this unique case presents a minimally invasive approach as an alternative in selected patients. This approach was chosen because it enables a smaller surgical incision, minimizes postoperative pain, promotes more rapid recovery, reduces wound-related morbidity, and offers technical advantages in the event of reoperation. A right mini-thoracotomy allowed safe exposure of the right atrium and tricuspid valve, enabling precise localization and controlled removal of the coil without damage to the valvular apparatus. The short cardiopulmonary bypass and cross-clamp times, combined with an uncomplicated postoperative course, further support the feasibility of this approach.

Literature concerning endovascular coil embolization to the right ventricle is scarce. A literature review by Leitman et al. on the migration of cardiac foreign bodies reported outcome results in 104 cases, dating from 1965 to 2014. The foreign objects included stents, catheters, metal fragments, vena cava filters, and different types of shunts, but no migration of coils was noted [9].

Rastogi et al. reported a case with migration to the right ventricle, near the tricuspid annulus. Percutaneous retrieval of the coil was not attempted because the coil seemed to be entrapped within the tricuspid annulus. Considering the small size of the coil (3 mm x 2 cm) and the minimal risk of further migration in the future, they opted to leave the coil in place. The patient remained asymptomatic during the two-year follow-up period [13]. In contrast to our case, where the migrated coil was significantly larger (1.5 cm x 1.5 cm), conservative management was not considered feasible due to the presumed increased risk of ventricular wall perforation, tricuspid valve injury, and infective endocarditis. However, no data are currently available that associate these complications with migrated coils in the heart. These concerns were based on reports of other types of intracardiac foreign bodies [16].

In cases of extracardiac coil migration, an endovascular approach is a suitable option. A recent study by Schechter et al. identified 19 case series between 2000 and 2012. A total of 574 cases of intravascular foreign bodies were reported in these series. Coil migration was mentioned in 17 patients. In 94% of cases, an endovascular retrieval was successful, with an additional 1.6% retrieved through a combined open and endovascular approach, and only 4% of objects were unretrieved using minimally invasive methods [11].

This case highlights several important clinical considerations. First, intracardiac coil migration, although rare, requires prompt recognition and thorough imaging to assess location, mobility, and degree of fixation. Second, management decisions should be guided by a multidisciplinary heart team, balancing the risks of intervention against the potential for long-term complications if the coil is left in situ. Finally, minimally invasive surgical extraction represents a safe and effective treatment option when endovascular retrieval is not feasible, particularly in cases involving valvular proximity or myocardial adherence.

## Conclusions

Migration of an endovascular coil to the right ventricle is a very rare complication of ovarian vein embolization. Optimal management requires multidisciplinary discussion to weigh the risks and benefits of conservative, endovascular, and surgical strategies. This case demonstrates that minimally invasive surgical extraction via right mini-thoracotomy can be performed safely and effectively in selected patients. A right mini-thoracotomy should be considered a viable alternative to median sternotomy when endovascular retrieval is not feasible, and coil removal is indicated.
